# Supplementary material for: Metabolic and Redox Pathway Dysregulation in HIV-Associated Coronary Endothelial Dysfunction: Insights into Early-Phase HIV Vascular Dysfunction
Source: bioRxiv. 2025 Dec 1:2025.11.26.690743. Preprint. [Version 1] doi: 10.1101/2025.11.26.690743 (PMC12694598; doi:10.1101/2025.11.26.690743)
Supplement: Supplement 2 [file media-2.pdf]

Supplementary Figure 2:

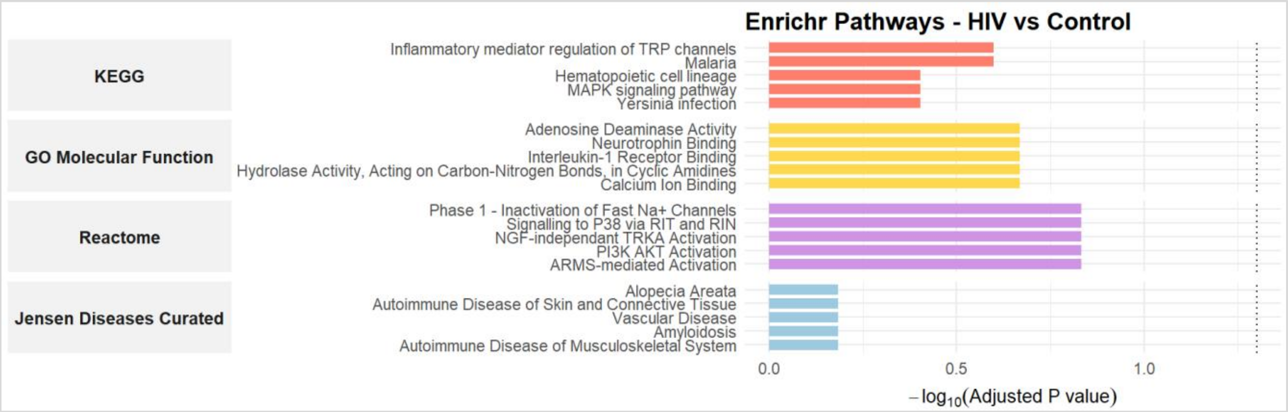

Supplementary Figure 2: Pathway analysis - Pathway enrichment analysis of differentially expressed proteins between normal and abnormal endothelial function groups with unadjusted p value < 0.005. The bar plot shows the top 5 enriched pathways according to KEGG, Gene Ontology (GO) Molecular Function, Reactome, and Jensen Diseases Curated databases. The x-axis represents the -$\log_{10}$(adjusted p value) for each pathway, with a higher value indicating stronger enrichment. Not a single pathway in any of these databases had an adjusted p-value of less than 0.05 which indicates that no pathways were enriched significantly. The black dashed line denotes the adjusted p-value threshold of 0.05.
