# Supplementary material for: Metabolic and Redox Pathway Dysregulation in HIV-Associated Coronary Endothelial Dysfunction: Insights into Early-Phase HIV Vascular Dysfunction
Source: bioRxiv. 2025 Dec 1:2025.11.26.690743. Preprint. [Version 1] doi: 10.1101/2025.11.26.690743 (PMC12694598; doi:10.1101/2025.11.26.690743)
Supplement: Supplement 3 [file media-3.pdf]

Supplementary Table 1: Additional characteristics of patients living with HIV

**Measure**	**Mean ± SD or %**
**CD4 count, cells/μL**	693.1 ± 375.7
**CD4 nadir, cells/μL**	336.0 ± 311.5
**CD4/CD8 ratio**	1.78 ± 2.25
**On ART**	98%
**Protease inhibitor use**	16%
**NNRTI use**	18%
**NRTI use**	73%
**INSTI use**	82%

Abbreviations: NNRTI = non-nucleoside reverse transcriptase inhibitor; NRTI = nucleoside reverse transcriptase inhibitor; INSTI = integrase strand transfer inhibitor.
