# Supplementary material for: Metabolic and Redox Pathway Dysregulation in HIV-Associated Coronary Endothelial Dysfunction: Insights into Early-Phase HIV Vascular Dysfunction
Source: bioRxiv. 2025 Dec 1:2025.11.26.690743. Preprint. [Version 1] doi: 10.1101/2025.11.26.690743 (PMC12694598; doi:10.1101/2025.11.26.690743)
Supplement: Supplement 4 [file media-4.pdf]

Supplementary Table 2. Baseline Characteristics by Coronary Endothelial Function (CEF) Status

<u>Variable</u>		HIV- (n = 29)	HIV+ (n = 45)	p-value
**Age, years (mean ± SD)**		52.4 ± 19.4	56.9 ± 12.4	**0.23**
**Female sex, %**		34	49	**0.22**
**Race, %**	**Black**	45	62	**0.14**
	**White**	34	31	**0.76**
	**Asian**	10	2	**0.13**
**Ethnicity, %**	**Hispanic**	10	7	**0.62**
	**Non-Hispanic**	90	91	
**BMI, kg/m² (mean ± SD)**		27.0 ± 7.6	29.3 ± 4.8	**0.12**
**Hypertension, %**		71	62	**0.56**
**Diabetes, %**		14	7	**0.35**
**Smoking history, %**		25	40	**0.20**
**Smoking, packs/day (mean ± SD)**		0.79 ± 0.62	1.02 ± 0.79	**0.49**

**History substance abuse, %**		26	43	**0.15**
**Total cholesterol, mg/dL (mean ± SD)**		176.0 ± 37.3	183.6 ± 42.3	**0.45**
**LDL cholesterol, mg/ (mean ± SD)**		96.4 ± 32.4	108.1 ± 39.2	**0.20**
**HDL cholesterol, mg/dL (mean ± SD)**		60.7 ± 18.9	53.8 ± 15.4	**0.096**
**Triglycerides, mg/dL (mean ± SD)**		98.0 ± 71.3	115.7 ± 52.6	**0.23**
**Abnormal coronary endothelial function, %**		10	67	**<0.001**
**% Change in Coronary Cross-Sectional Area (mean ± SD)**		4.28 ± 16.99	−2.99 ± 10.99	**0.037**
